# Assessing Endogenous and Exogenous Hormone Exposures and Breast Development in a Migrant Study of Bangladeshi and British Girls

**DOI:** 10.3390/ijerph17041185

**Published:** 2020-02-13

**Authors:** Renata E. Howland, Nicole C. Deziel, Gillian R. Bentley, Mark Booth, Osul A. Choudhury, Jonathan N. Hofmann, Robert N. Hoover, Hormuzd A. Katki, Britton Trabert, Stephen D. Fox, Rebecca Troisi, Lauren C. Houghton

**Affiliations:** 1 Department of Epidemiology, Mailman School of Public Health, New York, NY 10032, USA; rr2717@cumc.columbia.edu; 2 Yale School of Public Health, Yale University, New Haven, CT 06510, USA; nicole.deziel@yale.edu; 3 Division of Cancer Epidemiology and Genetics, National Cancer Institute, Bethseda, MD 20892, USA; hofmannjn@mail.nih.gov (J.N.H.); hooverr@exchange.nih.gov (R.N.H.); katkih@mail.nih.gov (H.A.K.); britton.trabert@nih.gov (B.T.); 4 Department of Anthropology, Durham University, Durham DH13LE, UK; g.r.bentley@durham.ac.uk; 5 Faculty of Medical Sciences, Newcastle University, Newcastle NE24HH, UK; mark.booth@ncl.ac.uk (M.B.); troisir@mail.nih.gov (R.T.); 6 Sylhet MAG Osmani Medical College, Sylhet WV23JH, Bangladesh; malwahouse@yahoo.com; 7 Cancer Research Technology Program, Leidos Biomedical Research, Inc., Frederick National Laboratory for Cancer Research, Frederick, MD 21702, USA; foxsd@mail.nih.gov

**Keywords:** cancer, adolescents, environmental exposure, estrogen, migrant study, BPA

## Abstract

Timing of breast development (or thelarche) and its endogenous and exogenous determinants may underlie global variation in breast cancer incidence. The study objectives were to characterize endogenous estrogen levels and bisphenol A (BPA) exposure using a migrant study of adolescent girls and test whether concentrations explained differences in thelarche by birthplace and growth environment. Estrogen metabolites (EM) and BPA-glucuronide (BPA-G) were quantified in urine spot samples using liquid chromatography tandem mass spectrometry (LC-MS/MS) from a cross-sectional study of Bangladeshi, first- and second-generation Bangladeshi migrants to the UK, and white British girls aged 5–16 years (*n* = 348). Thelarche status at the time of interview was self-reported and defined equivalent to Tanner Stage ≥2. We compared geometric means (and 95% confidence interval (CIs)) of EM and BPA-G using linear regression and assessed whether EM and BPA-G explained any of the association between exposure to the UK and the age at thelarche using hazard ratios and 95% confidence intervals. Average EM decreased with exposure to the UK, whereas BPA-G increased and was significantly higher among white British (0.007 ng/mL, 95% CI: 0.0024–0.0217) and second-generation British-Bangladeshi girls (0.009 ng/mL, 95% CI: 0.0040–0.0187) compared to Bangladeshi girls (0.002 ng/mL, 95% CI: 0.0018–0.0034). Two of four EM ratios (16-pathway/parent and parent/all pathways) were significantly associated with thelarche. The relationship between exposure to the UK and thelarche did not change appreciably after adding EM and BPA-G to the models. While BPA-G is often considered a ubiquitous exposure, our findings suggest it can vary based on birthplace and growth environment, with increasing levels for girls who were born in or moved to the UK. Our study did not provide statistically significant evidence that BPA-G or EM concentrations explained earlier thelarche among girls who were born or raised in the UK.

## 1. Introduction

Breast cancer incidence varies widely by country, with some of the highest rates in Western Europe and lowest in Asia [1]. For example, the age-standardized breast cancer incidence rates in 2018 were nearly six times higher in the United Kingdom (UK) (93.6 per 1,000,000) than in Bangladesh (17.0 per 1,000,000) [2]. Several epidemiological studies have focused on the role of endogenous hormone levels and breast cancer risk, and have used age at menarche as a proxy for the first dose of cumulative lifetime estrogen levels. However, other pubertal milestones, such as timing of onset of breast development (thelarche), which occurs before menarche, may be important indicators of estrogenic activity. The time period between thelarche and menarche may also be relevant for breast cancer risk because the length of time between these two milestones may indicate a sensitive time period when individuals are more susceptible to exposures to exogenous estrogenic chemicals. During puberty, the breast contains the highest number and greatest proliferative activity of the terminal duct lobular units [3], the most common site of ductal carcinoma [4]. Thus, early puberty, either as a proxy of endogenous exposure dose or a longer sensitive window to exogenous factors, may have direct or indirect effects on breast cancer risk later in life.

One hypothesized driver of earlier pubertal timing is exogenous exposure to hormonally active agents, commonly referred to as endocrine disruptors. Bisphenol-A (BPA) is one such agent that is thought to be ubiquitous in industrialized settings through its use in water bottles, food liners, and drinking water pipes, but relatively unknown in low-middle income countries [5]. In Bangladesh, consumption of bottled drinks has increased, potentially presenting an important source of BPA exposure [6,7]. Bangladesh was also the first country in the world to ban plastic bags, thus the level of exposure is unclear. Animal models suggest BPA demonstrates estrogenic activity [8], accelerates pubertal onset [9], and increases mammary cancer risk [10]. However, epidemiological evidence between BPA and puberty has been mixed [11]. Nevertheless, the World Health Organization commissioned a report in 2012 on endocrine-disrupting chemicals and recommended research should investigate whether early-life exposures are associated with changes in the timing of pubertal events [12].

Historically, studies of migrant populations have enabled examination of differences in environmental and social factors associated with breast cancer risk [13]. A recent example by Houghton et al. (2014) used a migrant study of British-Bangladeshi girls (named the Adolescence among Bangladeshi and British Youth project, or ABBY) to test whether the growth environment and/or ethnicity were associated with age and the tempo of pubertal milestones [14]. This population is unique in that 95% of Bangladeshi migrants to the UK come from the Sylhet region, in the northeast of the country, and tend to be similar ethnically and socioeconomically, providing a sample in which to contrast environmental and social exposures [15]. Houghton et al. (2014) found that while age at menarche was similar between groups, thelarche occurred between 1 to 2 years earlier among British-Bangladeshi and white British girls, compared to Bangladeshi girls still living in their home country. While differences in body mass index (BMI) between the groups partially explained this effect, much of the difference was left unexplained, suggesting there could be other social or environmental exposures driving the observed earlier thelarche. 

The first objective of this study then was to assess the relationship between birthplace and growth environment and both endogenous estrogen levels and exogenous BPA exposure within the same cohort of British-Bangladeshi girls. The second objective was to test whether estrogen and BPA concentrations could explain any of the association between exposure to the UK growth environment (migrant scale) and thelarche previously reported [14]. Given the widespread exposure to endocrine disruptors and the concern surrounding early breast development [12] and its potential association with breast cancer later in life [16], this study represents an important step towards characterizing the patterning of exogenous exposures and endogenous hormones during adolescence, and their impact on pubertal events.

## 2. Materials and Methods

### 2.1. Data Source

This analysis was conducted using ABBY data, a cross-sectional study of Bangladeshi, Bangladeshi migrants to the UK, and white British girls aged 5–16 years of age (*n* = 469), details of which are published elsewhere [15]. Briefly, girls were recruited from schools in London, England and Sylhet city, Bangladesh from September 2009 to April 2011. After obtaining written consent from parents and verbal assent from girls, researchers interviewed participants in person and collected anthropometric measurements and urine samples. The ABBY project received IRB approval from Department of Anthropology, Durham University Ethics Committee and the Sylhet M.A.G Osmani Medical College. Additional approval for the current analysis was obtained from the Columbia University Medical Center’s IRB (IRB-AAAS0007). 

### 2.2. Measures

Migrant scale: Participants were classified according to their birthplace, their parents’ birthplace, and self-reported ethnicity into the following categories: (1) Bangladeshi (those who were born and reside in Bangladesh), (2) first-generation British-Bangladeshi (those born in Bangladesh but residing in the UK), (3) second-generation British-Bangladeshi (those born in the UK to parents who emigrated from Bangladesh), and (4) white British (those who were born in the UK to parents of white British ethnicity). This scale captures increasing exposure to the UK environment, a proxy for a range of social and environmental exposures. 

Outcome: Thelarche status was assessed based on self-reporting using a modified version of the pubertal development scale (PDS), and was defined as the PDS equivalent to Tanner Stage 2 or higher [17,18]. Due to the cross-sectional nature of the data, thelarche was categorized as yes/no based on whether or not it had occurred at the age of interview. 

Urine Sample Collection: Urine spot samples were provided by UK and Bangladeshi participants between 9:00 and 16:00 h and placed directly on ice. The urine was aliquoted and stored at 20 °C. UK samples could be transported on ice to a local hospital for processing and temporary storage until samples were transferred to Durham University. Samples from Bangladesh were transported on ice to Sylhet M.A.G Osmani Medical College, where they were aliquoted and stored at −20 °C until being shipped on dry ice to the UK. All UK and Bangladesh urine samples were shipped on dry ice to a National Cancer Institute biorepository in the USA and then to the analytical lab. 

Estrogen Metabolites: The Cancer Research Technology Program, Frederick National Laboratory for Cancer Research conducted liquid chromatography tandem mass spectrometry (LC-MS/MS) on 0.5 mL of urine to measure 15 estrogens and estrogen metabolites (referred to collectively as EM). Details of this method have been published previously [19,20]. Briefly, conjugated (glucuronide and sulfate) EMs were hydrolyzed enzymatically and then measured together with unconjugated metabolites. EMs included parent estrogens (estrone and estradiol), 2 and 4-methylated and unmethylated catechols, and 16-hydroxylated metabolites. All coefficients of variation were below, or equal to, 3% for each estrogen using internal repeat quality control samples. 

Similar to previous studies, we examined EM in four groupings: (i) all EM combined, (ii) parent estrogens only (estrone and estradiol), (iii) the 2-, 4-, and 16-pathways, and (iv) ratios of those pathways [21]. The 2-pathway included 2-Hydroxyestrone, 2-Hydroxyestradiol, 2-Methoxyestrone, 2-Methoxyestradiol, 2-Hydroxyestrone-3-methyl ether; the 4-pathways included 4-Hydroxyestrone, 4-Methoxyestrone, 4-Methoxyestradiol; and the 16-pathways included 16α-Hydroxyestrone, Estriol, 17-Epiestriol, 16-Ketoestradiol, and 16-Epiestriol. The four ratios we examined were the 2-pathway to parent, 4-pathway to parent, 16-pathway to parent, and parent to 2-, 4-, 6-pathway.

Bisphenol-A: The same urine samples were used to estimate BPA exposure. The urine assay for BPA and BPA-G, the major conjugate excreted in urine, has demonstrated good reproducibility (Coefficient of Variation = 6.7%; Intraclass Correlation Coefficient = 99.5%, meaning there was low variation and high correlation between samples) [20], and is generally preferred over serum or plasma, due to the poor detectability in blood and potential contamination from blood collection materials [22]. In this study, collection tubes were known to be BPA-free. Since BPA is extensively metabolized to BPA-G and excreted, we focused on the conjugated form (BPA-G) because it avoids the potential for false values from background contamination. BPA-G values below the limit of detection (*n* = 67) were replaced as 0.01 ng/mL (the limit of detection). All EM and BPA-G values were divided by creatinine to correct for urinary dilution, log2-transformed to account for the skewed distribution, and added to models as continuous variables. We used log-base 2, rather than traditional log-base 10, to improve interpretability since the results can be interpreted as the effect per doubling of the hormone. Creatinine was measured by PPD © using an enzymatic colorimetric assay; all CVs were below 2%.

Other variables: At the time of urine collection, participant age was recorded and anthropometric measurements, including height, weight, and waist circumference were taken while the participant was clothed but without shoes. BMI was calculated in kg/m^2^.

Missing data: Of the 348 girls with urine samples, 22 (6.3%) were missing data on whether thelarche had occurred, 4 (1.6%) were missing age at urine sample collection, 39 (11.2%) were missing information to calculate BMI, and 24 (6.9%) were missing waist circumference. Additionally, 11% were missing BPA-G as a result of sample volume constraints. In order to explore the potential for selection bias, we examined differences between girls with and without urine samples, and between girls with and without BPA-G. Girls who did not provide urine were more likely to be Bangladeshi in Sylhet or to have a higher BMI (Appendix A Table A1). No significant differences were found between girls with BPA-G measurements and the total sample. The final study population included 348 girls. 

### 2.3. Statistical Analysis

To characterize differences in EM and BPA by migrant scale (objective 1), we estimated differences in the geometric mean and 95% confidence interval (CI) of urinary EM and BPA-G concentrations by the migrant scale using linear regression. Each of the 11 EMs and BPA-Gs served as the dependent variables and were modeled separately with the migrant scale as the primary predictor. Since age and BMI are strongly related to estrogen production and there were known differences in age and BMI between groups, we adjusted all EM models by age and BMI. Regression coefficients were back-transformed for presentation in the tables. In order to assess the impact of menstrual cycle day on urine collection, we performed an additional sensitivity analysis where we removed postmenarche girls (*n* = 72) from the models and compared results to the model with all girls included.

To test whether estrogen and BPA explained any of the associations between migrant scale and thelarche, we assessed whether the effect of migrant scale on thelarche changed after adjusting for EM or BPA-G by examining the percentage change in the beta coefficient. We used Weibull regression models, a parametric survival analysis [23], to account for both left censoring (for participants who reached thelarche before enrollment into the study) and right censoring (for participants who had not yet reached thelarche). More information on this method and how it compares with traditional Cox models can be found in Houghton et al. (2014) [14]. We estimated hazard ratios and 95% confidence intervals, interpreted as the risk of reaching thelarche at a given age. 

Statistical significance for all analyses was defined as *p* < 0.05. We used SAS 9.4 (Cary, NC, USA) for all analyses, except for the Weibull regression, which was conducted in STATA Version 15.0 (STATA Corporation, College Station, TX, USA).

## 3. Results

### 3.1. Sample Characterististics 

The study population included 161 Bangladeshi, 33 first-generation British-Bangladeshi, 114 second-generation British-Bangladeshi, and 40 white British girls, with an average age of 9.7, 11.5, 10.1, and 9.9 years, respectively (Table 1). Bangladeshi girls had the lowest average BMI compared with other groups, and the lowest average creatinine excretion (Table 1). Consistent with the age distributions of each group, a greater proportion of first-generation British-Bangladeshi girls in the sample had already reached menarche (48%), compared to 24% and 27% among the Bangladeshi and second-generation British-Bangladeshi girls, respectively, and 13% among white British girls.

### 3.2. EM

EMs were detected among all study participants. The sum of all EMs combined ranged from 8.6 to 14,719 ng/mL. Second-generation British-Bangladeshi and white British girls had significantly lower levels of 2-pathway (0.06 (95% CI: 0.03, 0.13) and 0.05 (95% CI: 0.02, 0.13), respectively) and 4-pathway (0.01 (95% CI: 0.00, 0.02) and 0.01 (95% CI: 0.00, 0.02), respectively) EMs compared to Bangladeshi girls (2-pathway = 0.08 (95% CI: 0.05, 0.13), 4-pathway = 0.01 (95% CI: 0.01–0.02)). Second-generation British-Bangladeshi also had several significantly different EM ratios than Bangladeshi girls, including the ratio of 2-pathway to parent, 4-pathway to parent, 16-pathway to parent, and parent to the 2-, 4-, 16-pathway. In our sensitivity analysis where we removed girls who had reached menarche, the point estimates were similar but no longer significant.

### 3.3. BPA-G

BPA-G was detected in nearly 80% of the total sample, with urine concentrations ranging from 0.0396 to 69.8 ng/mL. Bangladeshi and first-generation British-Bangladeshi girls were more likely to have concentrations below the limit of detection (34% and 22%, respectively) compared to second-generation British-Bangladeshi (4%) or white British girls (15%). BPA-G concentrations increased significantly (*p* < 0.0001 for trend) with duration of time in the UK, and were nearly 2 times greater in white British compared with Bangladeshi girls (Table 2). White British (0.007 ng/mL, 95% CI: 0.0024–0.0217) and second-generation British-Bangladeshi girls (0.009 ng/mL, 95% CI: 0.0040–0.0187) had significantly higher BPA-G than Bangladeshi girls (0.002 ng/mL, 95% CI: 0.0018–0.0034).

### 3.4. Thelarche

The parent EMs, pathways, and sum of all EMs appeared to be inversely, but not significantly, associated with the onset of thelarche. Among the four ratios of pathways and parents, the 16-pathway to parent ratio, and the parent to 2-, 4-, and 16-pathway (combined) were significantly associated with thelarche (Table 3). The 16-pathway to parent ratio was associated with a 32% reduction in the onset of thelarche (HR = 0.68, 95% CI: 0.48–0.95). Conversely, the parent to the combined 2-,4-,16- pathway ratio was associated with a 44% increase in thelarche (HR = 1.44, 95% CI: 1.03–2.01). BPA-G concentrations appeared positively associated with onset of thelarche, but the HR was not statistically significant (HR = 1.04, 95% CI: 0.97–1.11).

Adding the sum or individual EM or BPA-G concentrations to the model assessing the relationship between migrant scale and thelarche had minimal effects on the association (Table 4). For example, after adding the sum of all EMs to the model with migrant scale and BMI, the HR for thelarche changed from 1.76 to 1.72, from 1.23 to 1.20, and from 2.20 to 2.12 among first-generation, second-generation, and white British girls compared to Bangladeshi girls (Model 2 vs. Model 3). A similar lack of effect was seen with the other EM (data not shown). Likewise, when adding BPA-G concentrations to the model with migrant scale, the HR for thelarche changed from 1.72 to 1.78, from 1.18 to 1.24, and from 1.85 to 1.93 among first-generation, second-generation, and white British girls compared to Bangladeshi girls (Table 5, Model 6 vs. Model 5).

## 4. Discussion

In this study of adolescent girls in Bangladesh and the UK, we found there were differences in the urinary concentrations of EM and BPA-G by place of birth and growth environment. In particular, urinary BPA-G concentrations were higher among those living in the UK for longer periods (whether native-born or migrant), while EM concentrations were lower. Our findings represent an important step towards simultaneously characterizing endogenous estrogen hormones and exogenous BPA exposure among adolescents in Bangladesh, an area where little information about BPA exposure is known. While we found some differences in thelarche by EM pathway or route of metabolism, our study did not provide evidence to suggest that variation in EM or BPA-G explained differences in thelarche between groups reported in our prior publication [14]. 

Recent reviews have highlighted the paucity of information on BPA exposure from low-middle income countries, where packaged foods and beverages containing BPA have become increasingly common but regulation has lagged behind that of developed countries [5]. One of the few studies documenting urinary BPA concentrations in Asia identified detectable levels in 94.3% of samples and a geometric mean concentration of 1.20 ng/mL based on an LC-MS/MS assay. The authors reported a high degree of variation among the seven southeast Asian and Middle Eastern countries included in the study (China, India, Japan, Korea, Kuwait, Malaysia, and Vietnam), with the highest concentrations in Kuwait (3.05 ng/mL) and lowest in Japan (0.95 ng/mL) [24]. In the US population, Calafat et al. report that 92.6% of adults had detectable BPA levels in the 2003–2004 National Health and Nutrition Examination Survey (NHANES), with a geometric mean of 2.6 µg/L [25]. A subsequent study of adolescents aged 12–19 years with pooled NHANES data from 2003–2010 found a similar mean BPA exposure (2.64 ng/mL) to the adult population [26]. While variation in urine sampling, assays, and form of BPA (e.g., BPA vs. BPA-G) makes a direct comparison difficult, we found BPA-G concentrations were detectable in 80% of our sample, with lower levels (66%) among Bangladeshi girls. Mean BPA-G concentrations were also significantly lower among girls from Bangladesh compared to those in the UK, indicating that environmental exposure to BPA-G varied meaningfully between countries. BPA exposure in Bangladesh may be lower than the UK as a result of dietary behaviors, as has been shown in other populations where there is less use of plastic bottles and frozen meals [27] or greater country-wide regulations, such as a ban on plastic bags that went into effect in Bangladesh in 2002 [28]. 

Conversely, we found that EM levels decreased with duration of time in the UK, although not significantly. This runs counter to evidence from adult studies where urinary estrogen levels tend to be higher in Western countries. For example, total estrogen and EMs were three times higher among Asian-American women in the US (Chinese, Japanese, and Filipino) than in Shanghai-based Chinese women [29]. We also found that specific EM ratios were significantly associated with the timing of thelarche. In particular, the 16-pathway to parent ratio was associated with an increased probability of thelarche, as was the parent to the combined 2-, 4-, 16-pathway ratio. This might indicate that higher endogenous levels of parent estrogens, as opposed to their downstream pathways, play a role in breast development. The 16-pathway to parent ratio has been implicated in previous studies of breast cancer, increasing risk among premenopausal women by 61% [30]. Our findings suggest that the balance of parent and pathway estrogen metabolites are not only relevant later in life, but may also alter the timing of pubertal events. Future studies of breast cancer risk should consider measuring estrogen exposure during sensitive developmental windows across the life course.

Despite some differences in urinary concentrations of BPA and estrogens between the groups, our study found limited evidence that these concentrations explained the association between exposure to the UK environment and earlier thelarche. Evidence concerning the relationship between BPA and pubertal outcomes has been mixed [11]. Several toxicological studies have reported that early exposure to low doses of BPA altered the development of rodent mammary glands, which manifested from the time of exposure and was exacerbated at puberty and beyond [31]. In mouse models, BPA accelerates pubertal onset [9], shows estrogenic activity [4], and increases mammary cancer risk [10]. In a Chinese case-control study, serum BPA was higher in girls with precocious puberty compared to controls [14]. Other cross-sectional studies with US girls have found no association with urinary BPA and thelarche [15,16]. In 2003–2008, NHANES urinary BPA was not significantly associated with age of menarche [17]. It may be that previous epidemiologic studies have not replicated the findings from toxicological studies because they lack an adequate range in BPA exposure, were underpowered, or did not fully capture long-term exposure based on urinary measures. If BPA is in fact not driving thelarche, as these mixed findings could suggest, other established drivers of pubertal timing include genetics, birth weight, stress, nutritional status, and other environmental exposures. 

Our study has several limitations to note. First, the data were cross-sectional and thelarche, estrogen, estrogen metabolites, and BPA were only assessed at one point in time. While intraclass correlation coefficients for EMs are high, indicating good reproducibility [32], BPA varies greatly over time within an individual [33]. We addressed left/right censoring of thelarche by using Weibull regression models, which provide a valid estimate of the hazard of thelarche at a given age, but reduce the statistical power of our analysis. Secondly, we did not collect detailed information on all known drivers of estrogen, including sleep, and menstrual cycle day from girls who had reached menarche and cannot account for these sources of variability in estrogen levels. We had crude dietary pattern data, but not at the micronutrient level where we would expect to see effects on metabolism. We also had data on medication taken in the previous two weeks, which was minimal (<20%), and primarily pain killers. We also could not control for all potential sources of variability in urinary concentration of BPA. For example, individual differences in metabolism, such as the activity of glucuronosyl transferases or BPA conjugation, could affect the bioaccumulation of BPA-g in urine. As a result, our biomarker could reflect different lengths of exposure time; however, there is no reason to believe this would vary by migrant group. Our study design prioritized collecting data from a population that had migrated, which allowed us to contrast exposure levels by place of birth and growth environment, an important contribution to the literature. However, this resulted in a relatively small study population, particularly among first-generation, British-Bangladeshi girls. This led to wide confidence intervals around estimates and potentially underestimated differences in effects. Finally, due to the school setting from where girls were recruited, thelarche was defined via self-reporting rather than clinical examination. While outcome misclassification is possible, previous studies have found good concordance between both self-report and physical examination, as well as with basal hormone levels [34]. Unless there was systematic over- or under-reporting of thelarche by the migrant group, any misclassifications would have attenuated results.

## 5. Conclusions

In response to growing concerns about early-life exposure to endocrine disruptors and pubertal outcomes, we explored the relationship between BPA, estrogen metabolites, and thelarche in a unique, cross-sectional migrant study of adolescent girls from the UK and Bangladesh. By contrasting birthplace and growth environment between countries, we found a clear pattern of increasing BPA-G and decreasing estrogen/estrogen metabolite concentrations with duration of time in the UK. We also found that the onset of thelarche was associated with ratios of parent to pathway estrogen metabolites, particularly the 16-pathway, which has been implicated in breast cancer research. Ultimately, BPA-G and estrogen did not explain earlier onset of thelarche in white British girls, which could indicate that other environmental or social factors were at play, or that the current study was underpowered.

## Figures and Tables

**Table 1:** Characteristics of the study sample of British and Bangladeshi girls 5–16 years of age with urine samples, Adolescence among Bangladeshi and British Youth (ABBY) Project (*n* = 348).

Characteristics	Migrant Scale				Total
	Bangladeshi	First Generation	Second Generation	White British	
*N*	161	33	114	40	348
Age in years at sample collection, mean (std)	9.7 (3.0)	11.5 (3.2)	10.1 (2.7)	9.9 (2.6)	10.0 (2.9)
Body Mass Index, kg/m^2^, mean (std)	16.6 (3.5)	20.2 (4.6)	18.7 (3.7)	20.0 (3.7)	18.0 (3.9)
Creatinine, ng/mL, mean (std)	91 (53.9)	130 (120.4)	166 (80.6)	147 (80.3)	126 (81.7)
Included in BPA assay, *n* (%)	150 (93%)	27 (82%)	105 (92%)	26 (65%)	308 (89%)
Pubertal events					
Menarche, %	24%	48%	27%	13%	26%
Thelarche, %	39%	76%	56%	74%	52%
Pubarche, %	26%	41%	40%	43%	34%

**Table 2:** Mean concentration of urinary estrogen metabolites (ng/mg creatinine) and Bisphenol-A-glucuronide (ng/mg creatinine) by migrant scale among British and Bangladeshi girls 5–16 years of age, ABBY Project.

Estrogen Metabolites ^a,b^ (EMs) (*n* = 300)	Migrant Scale ^c^								
	Bangladeshi		First Generation		Second Generation		White British		Trend *p*-Value
	Geometric Mean and 95% CI	*p*-Value	Geometric Mean and 95% CI	*p*-Value	Geometric Mean and 95% CI	*p*-Value	Geometric Mean and 95% CI	*p*-Value	
All EM combined	0.18 (0.10–0.30)	REF	0.15 (0.06–0.38)	0.38	0.15 (0.07–0.32)	0.11	0.12 (0.05–0.30)	0.05	0.03
Parent estrogens	0.02 (0.01–0.03)	REF	0.01 (0.00–0.04)	0.37	0.01 (0.01–0.03)	0.47	0.01 (0.00–0.03)	0.10	0.16
*Pathways*									
2-Hydroxylation pathway	0.08 (0.05–0.13)	REF	0.07 (0.03–0.16)	0.35	0.06 (0.03–0.13)	0.05	0.05 (0.02–0.13)	0.03	0.01
4-Hydroxylation pathway	0.01 (0.01–0.02)	REF	0.01 (0.00–0.02)	0.36	0.01 (0.00–0.02)	0.03	0.01 (0.00–0.02)	0.04	0.01
16-Hydroxlation pathway	0.09 (0.05–0.15)	REF	0.08 (0.03–0.20)	0.49	0.07 (0.03–0.16)	0.09	0.06 (0.03–0.16)	0.07	0.03
*Ratios*									
2-Pathway/Parent estrogens	5.20 (4.06–6.67)	REF	5.35 (3.47–8.24)	0.77	4.54 (3.15–6.54)	0.02	5.05 (3.30–7.72)	0.74	0.10
4-Pathway/Parent estrogens	0.75 (0.57–0.98)	REF	0.77 (0.49–1.23)	0.75	0.64 (0.44–0.95)	0.02	0.74 (0.47–1.16)	0.90	0.11
16-Pathway/Parent estrogens	6.00 (4.77–7.54)	REF	6.40 (4.30–9.53)	0.45	5.37 (3.83–7.52)	0.04	6.03 (4.08–8.92)	0.94	0.20
Parent estrogens/2-, 4-, 16-Pathways	0.08 (0.07–0.10)	REF	0.08 (0.05–0.12)	0.61	0.09 (0.07–0.13)	0.02	0.08 (0.06–0.12)	0.95	0.13
									
BPA-G Analysis (*n* = 308)									
BPA-G	0.002 (0.0018–0.0034)	REF	0.005 (0.0016–0.0142)	0.148	0.009 (0.0040–0.0187)	<0.01	0.007 (0.0024–0.0217)	<0.01	<0.01

^a^ All values as standardized by creatinine, mean values are from linear regression analyses and are back-transformed from log-base 2. ^b^ Estrogen concentrations are adjusted for age and body mass index (BMI). ^c^ We tested the effect of migrant scale as a categorical variable and as an ordinal variable. In both cases, Bangladeshi was the reference category.

**Table 3:** Hazard Ratio (HR) and 95% confidence interval for onset of thelarche by estrogens and BPA among British and Bangladeshi girls 5–16 years of age, ABBY Project.

Estrogen Metabolites ^a,b^ (*n* = 300)	Hazard Ratio	Lower 95% CI	Upper 95% CI
Total	0.92	0.81	1.06
Parent estrogens	0.97	0.86	1.09
*Pathways*			
2-Hydroxylation pathway	0.90	0.79	1.04
4-Hydroxylation pathway	0.92	0.80	1.07
16-Hydroxlation pathway	0.91	0.75	1.10
*Ratios*			
2-Pathway/Parent estrogens	0.77	0.57	1.03
4-Pathway/Parent estrogens	0.87	0.66	1.15
16-Pathway/Parent estrogens	0.68	0.48	0.95
Parent estrogens/2-, 4-, 16-Pathways	1.44	1.03	2.01
BPA-G Analysis ^a,c^ (*n* = 299)			
BPA-G	1.04	0.97	1.11

^a^ All the EMs and BPA-Gs were included in the models as continuous variables. ^b^ Estrogen analysis excludes those missing information on thelarche, age, and BMI (*n* = 48). ^c^ BPA-G analysis excludes those missing information on thelarche and age (*n* = 9).

**Table 4:** Effect of EM on the association between migrant scale and risk of thelarche among British and Bangladeshi girls 5–16 years of age, ABBY Project, includes sample with EM (*n* = 300).

Migrant Scale	Model 1 ^a^		Model 2 ^a^		Model 3 ^a^	
	Crude		Model 1+ BMI		Model 2 + Total EM	
	HR	95% CI	HR	95% CI	HR	95% CI
Bangladeshi	REF		REF		REF	
First-generation	2.47	1.12–5.44	1.76	0.80–3.85	1.72	0.78–3.81
Second-generation	1.58	1.03–2.44	1.23	0.77–1.98	1.20	0.75–1.94
White British	2.92	1.59–5.35	2.20	1.15–4.21	2.12	1.09–4.10

^a^ Analysis excludes those missing information on thelarche, age, or BMI (*n* = 48).

**Table 5:** Effect of BPA on the association between migrant scale and risk of thelarche among British and Bangladeshi girls 5–16 years of age, ABBY Project, includes sample with BPA assay (*n* = 277).

Migrant Scale	Model 4 ^a^		Model 5 ^a^		Model 6 ^a^		Model 7 ^a^	
	Crude		Model 4 + BMI		Model 5+ BPA		Model 6 + Total EM	
	HR	95% CI	HR	95% CI	HR	95% CI	HR	95% CI
Bangladeshi	REF		REF		REF		REF	
First-generation	2.42	1.10–5.33	1.72	0.78–3.79	1.78	0.8–3.98	1.75	0.77–3.93
Second-generation	1.5	0.99–2.29	1.18	0.73–1.9	1.24	0.74–2.06	1.2	0.71–2.02
White British	2.65	1.38–5.11	1.85	0.89–3.84	1.93	0.91–4.09	1.85	0.86–3.95

^a^ Analysis excludes those missing information on thelarche, age, BMI, and BPA (*n* = 71).

## Appendix A

**Table A1:** Characteristics of girls with and without urine samples.

Characteristics	No Urine		Urine		Total	*p*-Value
	N	%	N	%	N	
Total	121	26%	348	74%	469	
*Migrant scale*						<0.01
Bangladeshi	30	16%	161	84%	191	
First Generation	14	30%	33	70%	47	
Second Generation	60	34%	114	66%	174	
White British	17	30%	40	70%	57	
*Pubertal outcomes*						
Pubarche	26	19%	109	81%	135	0.27
Thelarche	42	20%	169	80%	211	0.44
Menarche	27	23%	88	77%	115	0.95
	**Mean (std)**		**Mean (std)**			
Age, years	9.8 (2.9)		10.0 (2.9)			0.42
Waist Circumference (cm)	62.2 (10.7)		58.5 (10.3)			<0.01
Body Mass Index, kg/m^2^	55.9 (359)		18.0 (3.9)			0.06

## References

[1] Torre L.A., Islami F., Siefel R.L., Ward E.M., Jemal A. (2017). Global Cancer in Women: Burden and Trends. Cancer Epidemiol. Biomark. Prev..

[2] CANCER TODAY Data Visualization Tools for Exploring the Global Cancer Burden in 2018. https://gco.iarc.fr/today/home.

[3] Colditz G.A. (1995). Fat, estrogens, and the time frame for prevention of breast cancer. Epidemiology.

[4] Knight C.H., Sorensen A. (2001). Windows in early mammary development: Critical or not?. Reproduction.

[5] Baluka S.A., Rumbeiha W.K. (2016). Bisphenol A and food safety: Lessons from developed to developing countries. Food Chem. Toxicol..

[6] Mourshed M., Masud M.H., Joardder U.H.J. (2017). Towards the effective plastic waste management in Bangladesh: A review. Environ. Sci. Pollut. Res. Int..

[7] Islam M., Hossain S., Islam T., Iqbal S.A. Municipal Solid Waste Management in Sylhet City, Bangladesh. Proceedings of the WasteSafe 2017―5th International Conference on Solid Waste Management in South Asian Countries.

[8] Hewitt S.C., Korach K.S. (2011). Estrogenic activity of bisphenol A and 2,2-bis(p-hydroxyphenyl)-1,1,1-trichloroethane (HPTE) demonstrated in mouse uterine gene profiles. Environ. Health Perspect..

[9] Nah W.H., Park M.J., Gye M.C. (2011). Effects of early prepubertal exposure to bisphenol A on the onset of puberty, ovarian weights, and estrous cycle in female mice. Clin. Exp. Reprod. Med..

[10] Weber Lozada K., Keri R.A. (2011). Bisphenol A increases mammary cancer risk in two distinct mouse models of breast cancer. Biol. Reprod..

[11] Leonardi A., Cofini M., Rigante D., Lucchetti L., Cipolla C., Penta L., Espoisto S. (2017). The Effect of Bisphenol A on Puberty: A Critical Review of the Medical Literature. Int. J. Environ. Res. Public Health.

[12] World Health Organization (2013). State of the Science of Endocrine Disrupting Chemicals―2012.

[13] Nelson N.J. (2006). Migrant studies aid the search for factors linked to breast cancer risk. J. Natl. Cancer Inst..

[14] Houghton L.C., Cooper G.D., Bentley G.R., Booth M., Chowdhury O.A., Troisi R., Ziegler R.G., Hoover R.N., Katki H.A. (2014). A migrant study of pubertal timing and tempo in British-Bangladeshi girls at varying risk for breast cancer. Breast Cancer Res..

[15] Núñez-de la Mora A., Chatterton R.T., Choudhury O.A., Napolitano D.A., Bentley G.R. (2007). Childhood conditions influence adult progesterone levels. PLoS Med..

[16] Bodicoat D.H., Schoemaker M.J., Jones M.E., McFadden E., Griffin J., Ashworth A., Swerdlow A.J. (2014). Timing of pubertal stages and breast cancer risk: The Breakthrough Generations Study. Breast Cancer Res..

[17] Marshall W.A., Tanner J.M. (1969). Variations in pattern of pubertal changes in girls. Arch. Dis. Child.

[18] Petersen A.C., Crockett L., Richards M., Boxer A. (1988). A self-report measure of pubertal status: Reliability, validity, and initial norms. J. Youth Adolesc..

[19] Xu X., Veenstra T.D., Fox S.D., Roman J.M., Issaq H.J., Falk R., Saavedra J.E., Keefer L.K., Ziegler R.G. (2005). Measuring fifteen endogenous estrogens simultaneously in human urine by high-performance liquid chromatography-mass spectrometry. Anal. Chem..

[20] Fox S.D., Falk R.T., Veenstra T.D., Issaq H.J. (2011). Quantitation of free and total bisphenol A in human urine using liquid chromatography-tandem mass spectrometry. J. Sep. Sci..

[21] Houghton L.C., Sisti J.S., Hankinson S.E., Xie J., Xu X., Hoover R.N., Eliassen A.H., Ziegler R.G. (2018). Estrogen Metabolism in Premenopausal Women Is Related to Early Life Body Fatness. Cancer Epidemiol. Biomarkers Prev..

[22] Ye X., Sisti J.S., Hankinson S.E., Xie J., Xu X., Hoover R.N., Eliassen A.H., Ziegler R.G. (2012). Concentrations of bisphenol A and seven other phenols in pooled sera from 3–11 year old children: 2001–2002 National Health and Nutrition Examination Survey. Environ. Sci. Technol..

[23] Royston P. (2004). Flexible Parametric Alternatives to the Cox Model: Update. Stat. J..

[24] Zhang Z., Alomirah H., Cho H.S., Li Y.F., Liao C., Minh T.B., Mohd M.A., Nakata H., Ren N., Kannan K. (2011). Urinary bisphenol A concentrations and their implications for human exposure in several Asian countries. Environ. Sci. Technol..

[25] Calafat A.M., Ye X., Wong L.Y., Reidy J.A., Needham L.L. (2008). Exposure of the U.S. population to bisphenol A and 4-tertiary-octylphenol: 2003–2004. Environ. Health Perspect..

[26] McGuinn L.A., McGuinn L.A., Ghazarian A.A., Su J., Ellison G.L. (2015). Urinary bisphenol A and age at menarche among adolescent girls: Evidence from NHANES 2003–2010. Environ. Res..

[27] Park J.S., Kim S., Park M., Kim Y., Lee H., Choi H., Lim S. (2017). Relationship between dietary factors and bisphenol a exposure: The second Korean National Environmental Health Survey (KoNEHS 2012–2014). Ann. Occup. Environ. Med..

[28] Onyanga-Omara J. Plastic Bag Backlash Gains Momentum. https://www.bbc.com/news/uk-24090603.

[29] Moore S.C., Matthews C.E., Ou Shu X., Yu K., Gail M.H., Xu X., Ji B.T., Chow W.H., Cai Q., Li H. (2016). Endogenous Estrogens, Estrogen Metabolites, and Breast Cancer Risk in Postmenopausal Chinese Women. J. Natl. Cancer Inst..

[30] Eliassen A.H., Spiegelman D., Keefer L.K., Veenstra T.D., Barbieri R.L., Willet W.C., Hankinson S.E., Ziegler R.G. (2012). Urinary estrogens and estrogen metabolites and subsequent risk of breast cancer among premenopausal women. Cancer Res..

[31] Betancourt A.M., Wang J., Jenkins S., Mobley J., Russo J., Lamartiniere C.A. (2012). Altered carcinogenesis and proteome in mammary glands of rats after prepubertal exposures to the hormonally active chemicals bisphenol a and genistein. J. Nutr..

[32] Fuhrman B.J., Xu X., Falk R.T., Dallal C.M., Veenstra T.D., Keefer L.K., Graubard B.I., Brinton L.A., Ziegler R.G., Gierach G.L. (2014). Assay reproducibility and interindividual variation for 15 serum estrogens and estrogen metabolites measured by liquid chromatography-tandem mass spectrometry. Cancer Epidemiol. Biomark. Prev..

[33] Ye X., Wong L.Y., Bishop A.M., Calafat A.M. (2011). Variability of urinary concentrations of bisphenol A in spot samples, first morning voids, and 24-hour collections. Environ. Health Perspect..

[34] Shirtcliff E.A., Dahl R.E., Pollak S.D. (2009). Pubertal development: Correspondence between hormonal and physical development. Child. Dev..

